# Development of Lipid Nanoparticles Containing Omega-3-Rich Extract of Microalga *Nannochlorpsis gaditana*

**DOI:** 10.3390/foods11233749

**Published:** 2022-11-22

**Authors:** Cristina Blanco-Llamero, Ruth M. Galindo-Camacho, Joel Fonseca, Antonello Santini, Francisco J. Señoráns, Eliana B. Souto

**Affiliations:** 1 Department of Pharmaceutical Technology, Faculty of Pharmacy, University of Porto, Rua de Jorge Viterbo Ferreira, 228, 4050-313 Porto, Portugal; 2 Healthy Lipids Group, Departmental Section of Food Sciences, Faculty of Sciences, Autonomous University of Madrid, 28049 Madrid, Spain; 3 Department of Pharmacy and Pharmaceutical Technology, and Physical Chemistry, Faculty of Pharmacy and Food Sciences, University of Barcelona, 08028 Barcelona, Spain; 4 Unit of Synthesis and Biomedical Applications of Peptides, IQAC-CSIC, 08034 Barcelona, Spain; 5 Institute of Nanoscience and Nanotechnology (IN2UB), University of Barcelona, 08028 Barcelona, Spain; 6 Department of Pharmacy, University of Napoli Federico II, Via D. Montesano 49, 80131 Napoli, Italy; 7 REQUIMTE/UCIBIO, Faculty of Pharmacy, University of Porto, Rua de Jorge Viterbo Ferreira, 228, 4050-313 Porto, Portugal

**Keywords:** microalgae, *Nannochloropsis gaditana*, nutraceuticals, omega-3 lipids, lipid nanoparticles, factorial design

## Abstract

Microalgae are described as a new source of a wide range of bioactive compounds with health-promoting properties, such as omega-3 lipids. This biomass product is gaining attention mainly due to its potential to accumulate different compounds depending on the species and environment, and it has been commonly recognized as a valuable nutraceutical alternative to fish and krill oils. In this work, we obtained the extract of the microalga *Nannochloropsis gaditana*, selected on the basis of its content of eicosapentaenoic acid (EPA) and glycolipids, which were determined using GC-MS and high-performance liquid chromatography (HPLC), respectively. To develop an oral formulation for the delivery of the extract, we used a 2^3^ factorial design approach to obtain an optimal lipid nanoparticle formulation. The surfactant and solid lipid content were set as the independent variables, while the particle size, polydispersity index, and zeta potential were taken as the dependent variables of the design. To ensure the potential use of the optimum LN formulation to protect and modify the release of the loaded microalga extract, rheological and differential scanning calorimetry analyses were carried out. The developed formulations were found to be stable over 30 days, with an encapsulation efficiency over 60%.

## 1. Introduction

There is an increasing interest in the search for alternative and new biomasses for the obtention of valuable compounds using an environmentally friendly approach, following the principles of green chemistry and the circular economy. To this aim, the use of alternative extraction techniques, such as pressurized liquid extraction, which allow for traditional and hazardous solvents, such as chloroform and methanol, to be avoided, are also gaining attention, particularly when formulations are intended for oral administration [1,2,3,4]. Microalgae are one of the most promising alternative biomasses able to accumulate a wide range of valuable compounds, such as lipids, carotenoids, and vitamins, which are interesting due to their health-promoting properties, related to cardiovascular, neurological, and visual diseases [5,6,7,8,9,10,11,12,13,14]. A wide range of bioactive compounds can be obtained from microalgae depending on the species. For example, the microalga *Nannochloropsis gaditana* (*N. gaditana*) is able grow in wastewater and to accumulate large amounts of omega-3 lipids, including eicosapentaenoic acid (EPA) and lipid bioactive compounds, such as carotenoids. Indeed, *N. gaditana* is described as a great lipid producer, being the major component of this microalga [15,16,17,18]. The main advantages of microalgae as an alternative source of bioactive compounds, especially omega-3 lipids, include the substitution of traditional sources of omega-3, fish and krill, which contribute to the overexploitation of oceans and abusive fishing. Moreover, microalgae can be cultivated in wastewater, pools, and bioreactors, without competing with secondary plants for arable lands [19]. Indeed, different microalgae extracts/oils and biomasses have been extensively studied in terms of safety and have been approved for human consumption by the European Food Safety Agency (EFSA), which is a crucial step for their use in human nutrition [20,21]. Some of these species are *Chlorella vulgaris, Spirulina platensis, Nannochloropsis gaditana*, and *Tetraselmis chuii*. All these factors make these organisms a sustainable alternative source of bioactive compounds for nutraceuticals [22,23,24,25].

Ensuring the adequate intestinal delivery of bioactive compounds is among the crucial aspects of the formulation process, and LNs have advantages over conventional dosage forms (for example, capsules) in this field [26,27]. The physicochemical protection of bioactive compounds and, in the case of lipophilic compounds, the impact of formulation on their solubility in aqueous media are the two main mechanisms involved. Other advantages include improved stability, sustained release, and increased bioavailability due to their nano-size [28,29].

Lipid nanoparticles (LNs) are obtained from lipid materials with a melting point above 40 °C so that a solid matrix is produced to ensure a controlled release profile of the loaded bioactive. The biocompatibility of LNs intended for the oral administration of nutraceuticals is ensured, as the lipids used in their production are the same as those found in the composition of foods. Lipids are known as absorption enhancers, thereby contributing to the increased bioavailability of ingredients taken in the form of LNs [28,29,30,31,32].

Some of the lipids commonly used for oral LN formulations include triglycerides, such as tristearin, tripalmitin, and trilaurin; such lipids as Witepsol^®^ or SOFTISAN^®^ series, glyceryl behenate, and cetyl palmitate; and fatty acids, such as stearic and palmitic acid [28,29]. Previous studies described the production of LNs containing different drugs and bioactive compounds for oral delivery. For instance, LNs containing insulin and SOFTISAN^®^100 as a solid lipid were successfully produced using a modified solvent evaporation technique, confirming that all components were well-tolerated after oral administration, with no toxicity reported [33,34]. The bioactive compound, curcumin, which is characterized by a low bioavailability and poor aqueous solubility, strongly limiting its potential health benefits, was also encapsulated into LNs. The authors successfully produced curcumin-LN using Compritol^®^ as a solid lipid, evaluating in vitro drug release with promising results [35].

An experimental factorial design makes it possible to study the influence of the components of formulations (e.g., surfactants and lipids), set as independent variables, on the physicochemical characteristics of the produced LNs (e.g., size, polydispersity index, and zeta potential), usually set as the dependent variables. The purpose of this is to achieve optimal production conditions with the minimum number of experiments [36,37,38].

The use of factorial designs in oral LN formulation has been extensively reported in the scientific literature. Mucoadhesive and controlled-release LNs using trimyristin as a solid lipid were developed by studying the influencing variables using a factorial design, limiting the number of experiments and establishing the effect of multiple variables on the formulation properties, including size (z-Ave), polydispersity index (PI), and zeta potential (ZP) [36]. The production of lipid nanoparticles containing sucupira essential oil was also optimized by employing a 2^2^ factorial design, setting z-Ave, PI, and ZP as dependent variables. The optimized formulation was then characterized for the encapsulated efficiency and studied on Caco-2 cell lines, revealing an improved oral drug delivery [39]. Other examples include the study of the encapsulation of alpha-pinene in Imwitor^®^ 900 K-composed LNs, evaluated using an experimental factorial design [40,41].

Regarding omega-3 fatty acids, recent works have been performed to understand lipid nanoparticle production using omega-3 lipids. Shahparast et al. [42] improved the pharmacokinetic behavior of polyunsaturated fatty acid (PUFA) oxidation, enhancing oxidative stability when it was included into NLC composed of Precirol^®^ ATO5, stearin, and palmitic acid as solid lipids.

Therefore, these findings suggest that the use of LNs might be a potential formulation approach for the protection of a wide range of bioactive compounds, including omega-3 lipids. However, due to the complexity of microalgae composition, further studies need to be performed to determine the optimal LN dispersion.

The present study aimed to produce and optimize omega-3 microalga extract lipid nanoparticles based on an experimental 2^3^ full factorial design. We carried out an assessment of the long-term stability of the LN formulations with different concentrations of solid lipid and surfactant by the variation in LN physicochemical properties (mean particle size, polydispersity index, and zeta potential).

## 2. Materials and Methods

### 2.1. Materials

*N. gaditana* dry biomass was obtained from AlgaEnergy (Madrid, Spain). The *N. gaditana* extract was obtained and analyzed in terms of lipid classes and fatty acid profile following previously optimized methods [1]. SOFTISAN^®^ 649 (bis-diglyceryl Polyacyladipate-2), a lanolin substitute widely use in the industry [43], was obtained from Sasol Germany GmbH & Co (Witten, Germany). Tween^®^ 80 (polysorbate 80) was purchased from Sigma Aldrich (Madrid, Spain). Soy lecithin was purchased from Acopharma (Barcelona, Spain). Ethyl acetate was from Vaz Pereira S.A. (Sintra, Portugal). MilliQ water was obtained from a MilliQ plus system (Millipore, Germany). Methanol was purchased from Lab Scan Analytical Sciences (Gliwice, Poland). The HPLC-grade solvents (2,2,4-trimethyl pentane, methyl tert-butyl ether (MTBE)) were purchased from Macron Fine Chemicals (Gliwice, Poland). Absolute ethanol (PRS grade), sodium hydrogen carbonate, and potassium hydroxide were purchased from Panreac Química S.A (Barcelona, Spain). Glyceryl trilinoleate, dioleoylglycerol (mixture of 1,3- and 1,2-isomers), 1-oleoyl-rac-glycerol, phosphatidylethanolamine, mono galactosyl glycerol, di galactosyl glycerol, oleic acid, and ethyl linoleate used as HPLC standards were purchased from Sigma-Aldrich (St. Louis, MO, USA). All other reagents and solvents used were of analytical or HPLC grade.

### 2.2. Factorial Experimental Design for LN Production

A factorial design method was used to maximize experimental efficiency, requiring the minimum number of experiments to optimize the LN formulation. Two independent variables and their influence on the physicochemical properties of the produced nanoparticles were studied using a 2k factorial design model composed of 2 independent variables with 3 levels each, as shown in Table 1. For each factor, the upper, middle, and lower levels were represented as (+1), (0), and (−1), respectively. These were selected based on previous pre-formulation studies and the available literature. The independent variables were the concentration of SOFTISAN^®^ 649 as a solid lipid (SL) in the formulation and the concentration of Tween^®^ 80. In this case, SOFTISAN^®^ 649 was chosen as the solid lipid because of its superiority over other lipid excipients in omega-3 formulations due to its food-grade classification and its low melting point, which make it possible to avoid high temperatures and protect the omega-3 extract during the process.

The dependent variables established were mean particle size, polydispersity index (PI), and zeta potential (ZP). Lecithin content was not studied as an independent variable, as its content in this production method was optimized previously in other works carried out by the research group [33]. Experiments were performed in triplicate, requiring a total of 15 experiments. This design allows for the optimization of methods by using different mathematical and statistical models, as response surface models and pareto charts are used to study the influence of the independent variables on the dependent ones.

### 2.3. Preparation of Nanoparticles

LN dispersions were prepared using a solvent evaporation method [33,36] in which, first, 5 mL of ethyl acetate extract of *N. gaditana* (mass concentration, 4:1), SOFTISAN^®^ 649 (1.25%), and soy lecithin (0.125 mg) were homogenized for 10 min in an Ultra-TurraxT25 at 40 °C (IKA^®^-Werke GmbH & Co, Staufen, Germany). This mixture was poured into 40 mL of MilliQ water containing Tween^®^ 80 and homogenized for another 5 min. Ethyl acetate was evaporated on a rotary evaporator (Buchi R-210, Merck Life Science, Algés, Portugal) for 30 min at 25°C. The temperature was controlled throughout the process to guarantee the stability of the *N. gaditana* extract. Finally, the particles were stored at 4 °C under refrigeration.

### 2.4. Particle Size and Zeta Potential

The particles were physicochemically characterized using a dynamic light scattering (DLS) analysis (NanoBrook Omni, Brookhaven Instruments, Holtsville, NY, USA). The samples were diluted in MilliQ water 1:10 *v*/*v* and analyzed at 20 °C, with a refractive index of 1.331 and a dielectric constant of 80.37. All mean particle size, PI, and ZP measurements were performed in triplicate.

### 2.5. Determination of Encapsulation Efficiency Using HPLC-ELSD

The encapsulation efficiency (EE) was calculated using the indirect method (Equation (1)), in which the non-encapsulated bioactive (glycolipids) was separated from the LNs via centrifugation for 5 min at 5000 G. The resulting supernatant corresponding to the non-encapsulated glycolipids was analyzed using HPLC-ELSD, and the encapsulation efficiency (EE%) was calculated according to Equation (1). Both the method and the HPLC-ELSD equipment were those already described.

(1) $$EE\left(\%\right)=\left(\frac{Glycolipids\;in\;the\;crude\;N.gaditana\;extract-Glycolipids\;in\;the\;supernatant}{Glycolipids\;in\;the\;crude\;N.gaditana\;extract}\right)100$$

### 2.6. Rheological Analysis

Rheology studies were performed on a Malvern Kinexus rheometer (Malvern Instruments, England). In this case, the oscillation frequency sweep test was applied in a range from 0 to 10 Hz. The storage modulus (G′), the loss modulus (G″), and the complex viscosity (η*) of the lipid nanoparticles were described as a function of frequency at a constant strain amplitude of 5 Pa (linear viscoelastic region). All measurements were carried out directly at room temperature (25 °C).

### 2.7. Differential Scanning Calorimetry

A differential scanning calorimetry (DSC) analysis was performed using a Mettler Toledo DSC 200 F3 System (NETZSCH-Gerätebau GmbH, Germany). Approximately 1–2 mg of bulk lipid, an equivalent LN dispersion containing a similar amount of the lipid, or *N. gaditana* extract was filled into 40 μL aluminum pans and sealed. The scan rate was 5 °C/min in the temperature range of 25–200 °C (above the melting point of each product), and each product was cooled down to 10 °C. An empty pan was used as a reference. Melting points correspond to the peak maximum of the heating curves.

### 2.8. Stability Analysis

The stability of the formulations was studied over 30 days, keeping samples stored at 4 °C in the dark, inside glass containers. LNs were analyzed on day 0, day 15, and day 30 in terms of PZ, IP, and Ave-z to study the stability of these parameters over time and to study the optimized formulation in comparation with the other samples.

### 2.9. Statistical Analysis

STATISTICA 7.0 software (Stafsoft. Inc., Moscow, Russia) was used for the response surface methodology (RSM) and to statistically analyze the data obtained.

## 3. Results

### 3.1. Chemical Composition of N. gaditana Extract

*N. gaditana* extract was characterized in terms of lipid composition in a previous work [1]. The HPLC analysis revealed a composition formed by different lipid species, such as triglycerides (TGs), diglycerides (DGs), free fatty acids (FFAs), monoglycerides (MGs), and glucolipids (GLs), where the main lipid class was GLs, which is in agreement with previous works on *N. gaditana* composition [44,45]. The fatty acid profile of the lipid class analyzed was investigated using a GC-MS analysis, showing a relative EPA content of 37.47%. Thus, *N. gaditana* extract can be considered an alternative source of PUFA omega-3 lipids, concretely, EPA. However, the main identified fatty acids were myristic acid (14:0), palmitic acid (16:0), palmitoleic acid (16:1), stearic acid (18:0), oleic acid (18:1), linoleic acid (18:2), linolenic acid (20:3), and eicosapentaenoic acid (20:5). Other fatty acids, such as DHA, were not found in these samples of *N. gaditana*. EPA was the main omega-3 fatty acid in the extract, as described by other authors [44,45,46].

### 3.2. Influence of Formulation Variables on the Production of LN Dispersions Containing N. gaditana Extract

**Figure 1:**
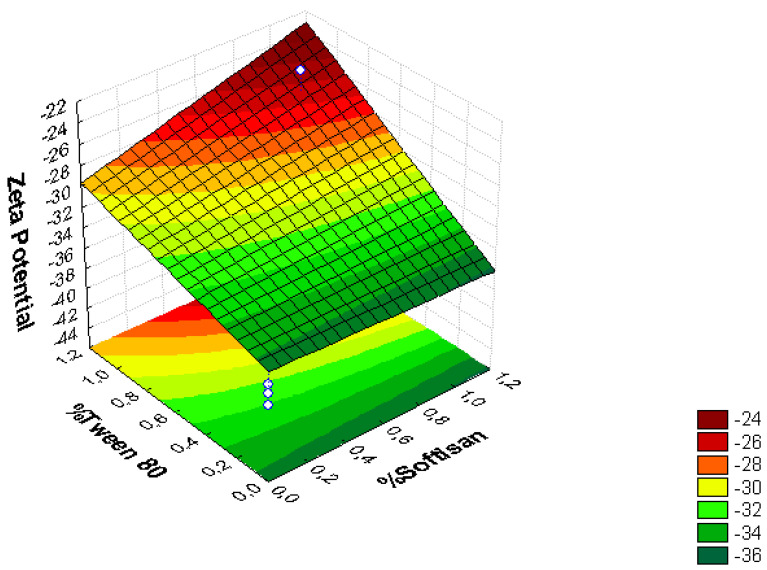
Surface response chart of (A) the effect of the % of Tween^®^ 80 and SOFTISAN^®^ 649 on ZP.

**Figure 2:**
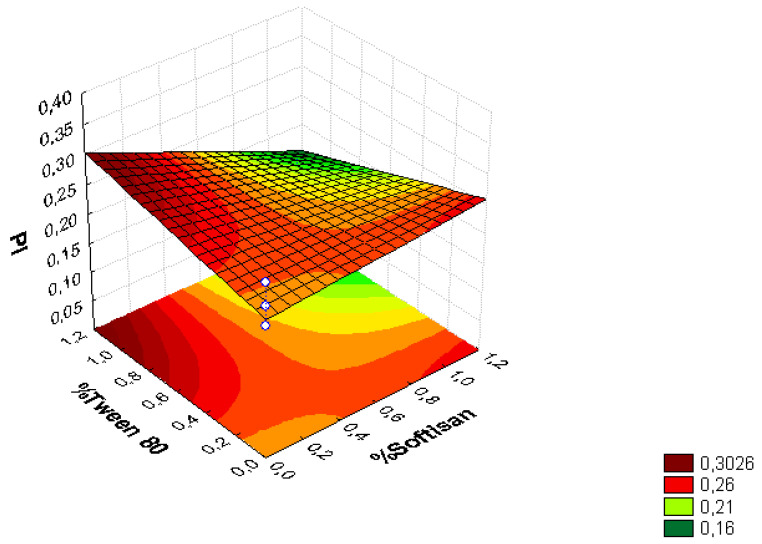
Surface response chart of the effect of the % of Tween^®^ 80 and SOFTISAN^®^ 649 on PI.

**Figure 3:**
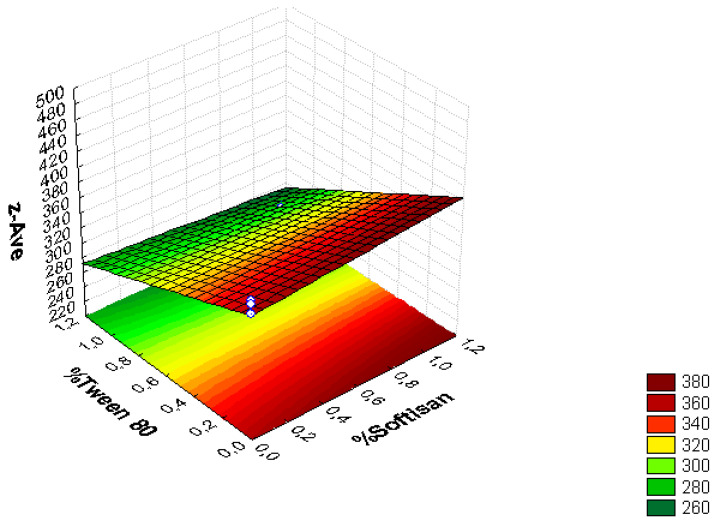
Surface response chart of the effect of the % of Tween^®^ 80 and SOFTISAN^®^ 649 on z-Ave.

**Figure 4:**
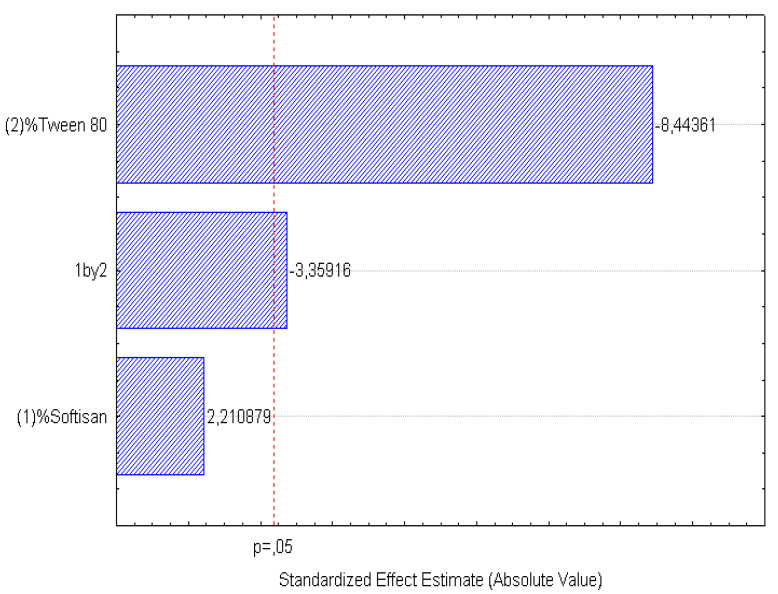
Pareto chart of the analyzed effects for z-Ave.

**Figure 5:**
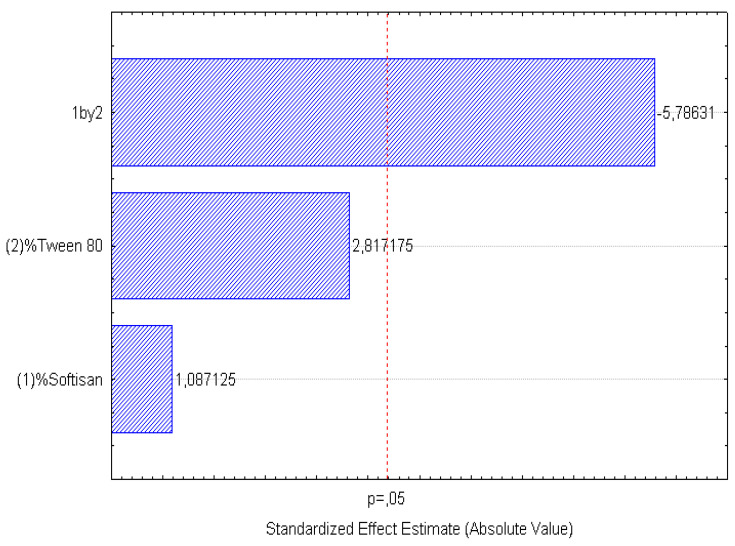
Pareto chart of the analyzed effects for PI.

**Figure 6:**
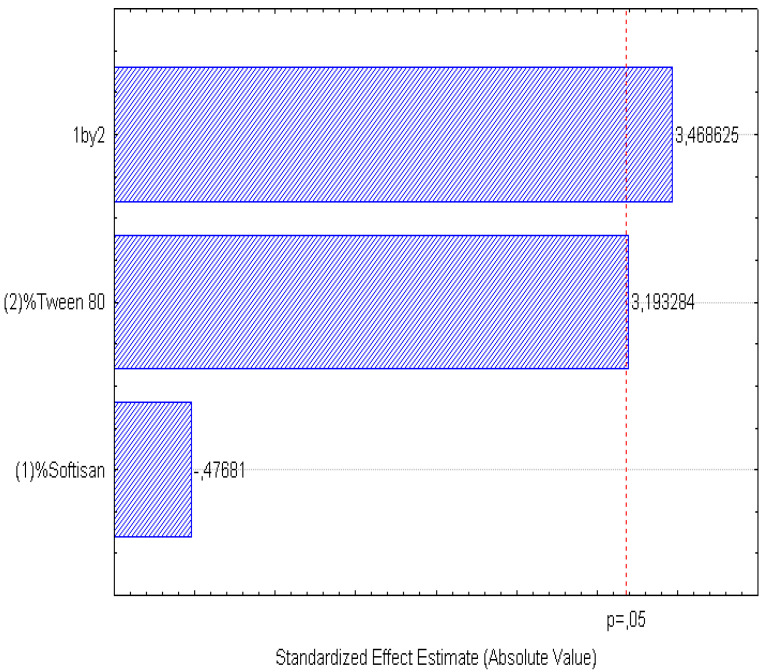
Pareto chart of the analyzed effects for ZP.

A factorial design was used to optimize the formulation of the LNs, and the obtained results are presented in Figure 1, Figure 2, Figure 3, Figure 4, Figure 5 and Figure 6, analyzing the effect of the independent variables (surfactant and solid lipid concentrations) on the dependent variables (Z_ave_, PI, and ZP).

From the surface response charts, it can be inferred that the independent variables, the % of solid lipid (SOFTISAN^®^ 649) and the surfactant (Tween^®^ 80), are directly related to the dependent variables, particle size, polydispersity index (PI), and zeta potential (ZP).

In the surface response graphs (Figure 1, Figure 2 and Figure 3) and in the Pareto diagram obtained for each dependent variable (Figure 4, Figure 5 and Figure 6), the significant influence of the interaction of the concentration of SOFTISAN^®^ 649 (X1) and that of Tween^®^ 80 (X2) on the three dependent variables (*p* < 0.05) is shown. In the case of PI and particle size, a negative effect of the interaction of the two factors was observed, meaning that there was an inverse proportional relationship between them. In fact, an increase in the percentages of X1 and X2 led to a smaller particle size and PI. Furthermore, Tween^®^ 80 had a significant influence on the particle size, being proportionally inverse. However, in the case of the zeta potential, a direct proportional relationship was observed, in which an increase in the two factors also increased this dependent variable.

The particle size, ZP, and PI expected for the optimized formulation, then, should ideally be <200 nm in size with a relatively low PI (<0.2) and ZP values > |20|. Taking into account which values of the physicochemical parameters were the objective, the optimum conditions for the independent variables were determined based on the presented plots.

From the results shown, it can be inferred that the particle size and the PI are the limiting factors of the formulations, taking 0.6% SOFTISAN^®^ 649 and 1% Tween^®^ 80 as the optimum conditions based on the response surface plots. The physicochemical characteristics of the particles obtained coincided with the expected results. The particle size was around 180 nm, with an PI of 0.13 and a ZP of −30. The encapsulation efficiency was found to be 61.48%, indicating an acceptable EE of the glycolipids from the *N. gaditana* extracts. Six different formulations (LN1-LN6), including the ones studied in the factorial design and that selected as the optimum (LN6), were prepared and tested to determine the stability of the dispersions.

### 3.3. Determination of the Stability of Dispersions by Means of Their Characterization Using DLS

To study the stability of the produced systems, the physicochemical characteristics of the particles were investigated over time for 30 days using DLS equipment. The DLS analysis was performed on the six types of LNs, and further analyses were performed on a chosen formulation (LN6). The compositions of the formulations studied along with the optimal LN formulation (LN 6) were as follows: LN 1 at 3% SL and 1% Tween^®^ 80, LN 2 at 3% SL and 0.1% Tween^®^ 80, LN 3 at 2.5% SL and 0.5% Tween^®^ 80, LN 4 at 1% SL and 1% Tween^®^ 80, and LN 5 at 1% SL and 0.1% Tween^®^ 80. Table 2 shows the results of the analysis on the day of production, on day 14, and on day 30 after production. The mean diameter measurements varied from 180.6 to 562.6 nm, PI varied from 0.05 to 0.523, and ZP varied from −44 to −20 mV, depending on the conditions used and the day of analysis. These results are in the range found previously for LNs containing nutraceuticals [42,47].

### 3.4. Determination of the Rheological Properties of the LN Obtained under Optimized Conditions (LN6)

**Figure 7:**
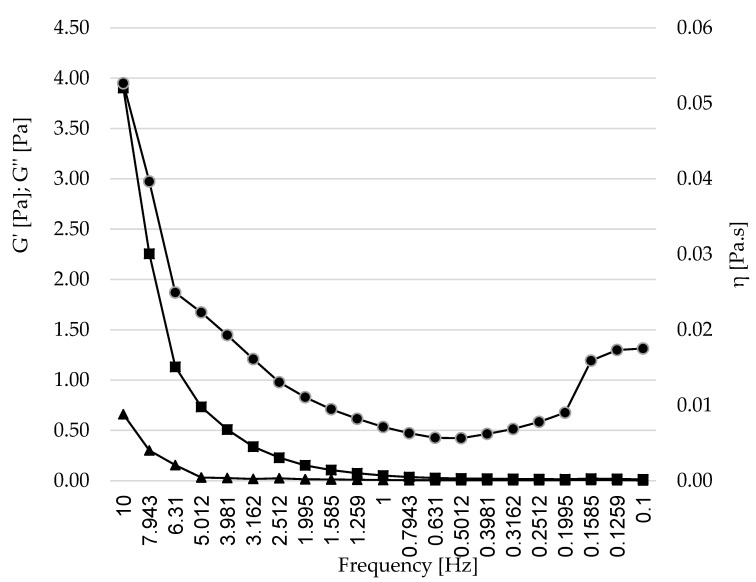
Rheological behavior of the LN6 dispersion (containing 0.6% of SL and 1% of Tween^®^ 80). Captions: Captions: storage modulus G′ (◼, viscous component), the loss modulus G″ (∆, elastic component), and η (●, complex viscosity).

An important part in the design and production of LNs is the behavior after in vivo administration or after inclusion in food matrices. This behavior can be predicted by studying the rheological properties of the formulation. Rheological features have a strong impact on physical and sensory characteristics of food systems [48,49]. Figure 7 shows the flow curves of the oscillation frequency sweep test of the aqueous LN dispersion at room temperature. The storage modulus G′ (viscous component), the loss modulus G″ (elastic component), and complex viscosity (η) are presented.

### 3.5. Differential Scanning Calorimetry Analysis

**Figure 8:**
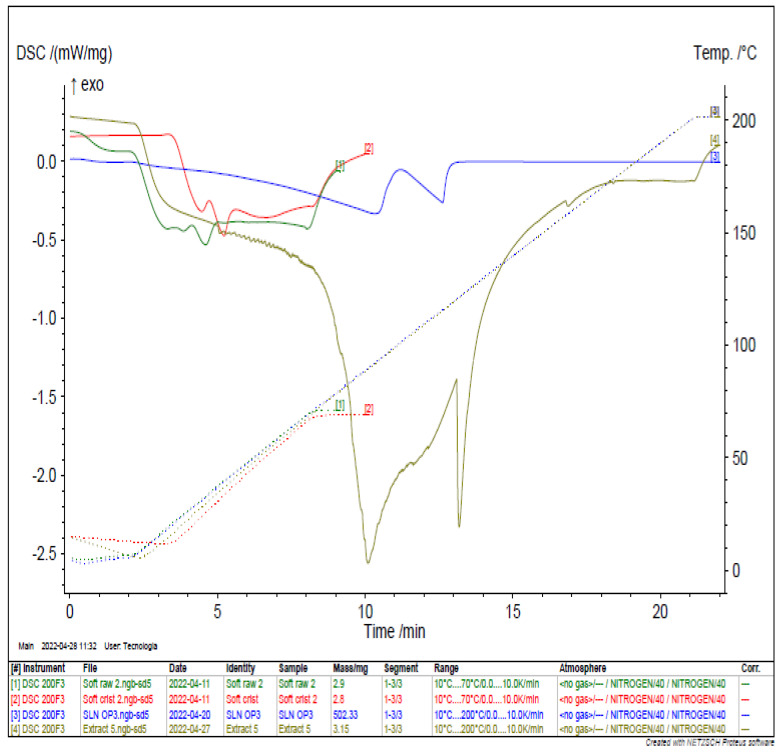
DSC analysis of the optimal LN (blue), *N. gaditana* extract (gray), raw SOFTISAN^®^ 649 (green), and crystallized SOFTISAN^®^ 649 (red).

The thermal behavior of the *N. gaditana* extract, raw SOFTISAN^®^ 649, recrystallized raw SOFTISAN^®^ 649, and the LN6 were determined using differential scanning calorimetry as shown in Figure 8 [50]. This well-established technique provides information on the melting and crystallization behavior of crystalline materials, such as lipid nanoparticles, to characterize physical and chemical changes in their enthalpy or the heat capacity of the lipid [51]. The raw and recrystallized SOFTISAN^®^ 649 thermograms showed endothermic melting peaks of 33.0 and 33.3 °C with enthalpies of −0.5312 mW mg^−1^ and −0.4742 mW mg^−1^, respectively. These values correspond to those expected from the literature review, with a slight variation, which may be due to the sensitivity of the instrument. However, the *N. gaditana* extract revealed two endothermic peaks at 89.4 °C and 121.1 °C with transition energies due to melting followed by decomposition and with enthalpies of −2.559 mW mg^−1^ and −2.33 mW mg^−1^, respectively. The extract was loaded into the lipid matrices to formulate nanoparticles. The LN sample showed two endothermic peaks at 92.1 °C and 115.8 °C, with corresponding enthalpies of −0.3344 mW mg^−1^ and −0.2631 mW mg^−1^, respectively.

## 4. Discussion

The analysis of the *N.gaditana* extract confirmed a fatty acid profile rich in EPA and glycolipids as being the main lipid class, which is in agreement with previously published works [44,45,52,53,54].

Taking into account the dependent variable of particle size, theoretically, an increase in the formulation of the solid lipid will affect the stability of the emulsion, giving rise to a higher particle size of the system. This fact favors the agglomeration of the particles, giving rise to a larger particle size and polydispersity index. To avoid this phenomenon, an increase in the percentage of the solid lipid should be accompanied by an increase in the concentration of the surfactant, as shown in Figure 1, Figure 2, Figure 3, Figure 4, Figure 5 and Figure 6. However, the crystallization phenomenon depends mainly on the concentration of the lipid and surfactants in the LN and may lead to the formation of micelles in the external aqueous phase of the emulsion [38]. These micelles normally have a size between 10 and 100 nm and may affect the polydispersity of the particle size of a system that has larger sizes [38,55]. Therefore, both phenomena must be taken into account when studying the concentrations of the independent variables in the system. LNs should ideally be <200 nm in size with a relatively low PI (<0.2) to be able to be administered intravenously, or pulmonary, for example, whereas larger sizes can be administered orally [28,32]. For oral administration, nano-sized particles improve the bioavailability of some bioactive compounds due to their increased surface area. Moreover, by decreasing the particle size, the thickness of the diffusion layer is decreased, promoting faster transport and absorption. Thus, low sizes for oral intake are also desirable [56]. Recent works on food nanotechnology reported a nanocarriers’ particle size from 100 to 500 nm with increased release and bioavailability of the compounds studied [42,47,57].

In contrast, ZP predicts the stability of the system, as well as the adhesive qualities of the particles, leading to a longer residence time at the site of action. In theory, high ZP values, whether positive or negative, tend to stabilize NP systems, so suspensions with ZP values > |20| promote the electrostatic repulsion of particles with the same electrical charge, thus avoiding particle aggregation and favoring system stability [30,37]. The results found in the present work were within the range described as optimal for this factor, finding negative values from −25 to −50 mV, which is characteristic of lipid nanoparticles with values that provide adequate system stability. After analyzing the trends obtained, the optimized formulation was developed and characterized containing 0.6% of SOFTISAN^®^ 649 and 1% of Tween^®^ 80. Further studies will be performed to study the influence of the extract amount in the formulation. Moreover, zeta potential experiments at different pH conditions should be carried out to observe how the zeta potential changes with reference to pH.

The encapsulation efficiency of the optimized LN containing the microalga extract was 61.48 ± 0.6%, showing results higher than previous ones in the production of lipid nanoparticles containing omega-3 oil, where the EE did not exceed 30 ± 0.4% [42]. However, the EE was lower than that found in other works producing LNs containing different nutraceuticals, where the EE was >90% [30,39]. These results show the potential of this work and the need to further investigate new materials for microalgae–LN formulations.

Taking into account day 0 (production day), Table 2 shows the different physicochemical characteristics of the obtained formulations. The influence of the % of SL along with the % of Tween^®^ 80 in the formulations on the particle characteristics is shown, where large nanoparticles were found when maintaining the same %SL and when the %surfactant employed was lower (LN 2 and 4). Taking into account day 14, the particle characteristics hardly changed from those on day 0. The particle size, ZP, and PI remained stable for all formulations, with slight variations per week of production. However, 30 days postproduction, a notable increase in particle size was observed for most of the formulations obtained. This increase was observed in every sample, but it was especially emphasized in the LN1 formulation, which corresponded to the lowest zeta potential on the day of production, affecting its stability and favoring the aggregation of particles and increasing their size, with a corresponding increase in the polydispersity of the formulation. This phenomenon was seen to a lesser extent in formulations LN 5 and 6, which correspond to those particles with a higher zeta potential. Thirty days after production, the optimal formulation (LN 6) kept the ZP relatively constant (−38 mV), but had a 0.85 times increased diameter size and a 0.86 times increased PI. 

For oral formulations, the knowledge of their rheological properties is critical in order to predict their behavior when included in food matrices or their mechanical interaction with gastric mucous [58,59]. With the purpose of evaluating the viscoelastic behavior of LN 6, dynamic oscillatory tests were performed. Previous works on nanoparticles for oral delivery have established that, ideally, LN dispersions should have a pattern where the storage modulus G′ (elastic component) and the loss modulus G″ (viscous component) rise with the applied frequency. Moreover, G′ should be greater than G″, indicating stronger structures that are more resistant against deformation, therefore contributing to the elastic behavior of the formulation [60,61,62]. There is an ascending pattern of G′ and G″, where G′ > G″, as shown in Figure 7, coincides with the ideal behavior exposed. The storage modulus G′ was greater than the loss modulus G″ over the measured frequency range, indicating the presence of a gel-like structure. The loss modulus G″ showed less dependence on the applied force, whereas the storage modulus G′ significantly increased over the frequency range from 0.1 to 10 Hz, highly depending on it. Some studies employing mucin models for the evaluation of mucoadhesiveness have shown that compounds with viscoelastic profiles with moduli G′ and G″ being frequency-dependent, where G″ is greater than G′ at a low frequency—and this value is inverted at high frequencies—have a behavior related to weak structure, suggesting poor interactions with mucin (less mucoadhesion) compared to products that maintain a constant viscoelastic profile with G′ greater than G″ [58,63]. However, the rheological analysis of the nanoparticles showed a behavior with a very low viscosity (η), which decreased with the applied frequency, demonstrating that the behavior is ideal, as based on previous works on LN dispersions [55,64,65]. However, it showed weak dependence on the applied frequency.

The results show the obtention of LN dispersions with appropriate consistency in a one-step production process without the need for further ingredients or stages. The DSC thermograms and the endothermic events of the main raw materials used to formulate the LNs, as well as the extract, are shown in Figure 8. The results demonstrate that the melting enthalpy decreased sharply when comparing the primary lipid and the LNs [66,67]. This decrease in enthalpy indicates a decrease in the lipid matrix crystallinity, and it is commonly reported in works on the production of nanocarriers with crystalline structures, such as lipid nanoparticles [68,69]. It has been reported that the presence of a drug/compound in the lipid matrix can generate alterations in the crystalline order of the lipids; thus, the encapsulation of the extract may play a significant role in the occurrence of this phenomenon [70,71]. Furthermore, the presence of surfactant in the formulation could be considered an additional reason for matrix crystallinity distortion and, subsequently, melting point variation [72,73].

## 5. Conclusions

Lipid nanoparticles (LNs) containing the extract of the microalga *N. gaditana* were successfully produced with an EE above 60%. The microalga extract was mainly composed of polar lipids, concretely, glycolipids containing 37.47 % of EPA. The factorial design of the experiments revealed an inverse relationship between the interaction of the concentration of Tween^®^ 80 and SOFTISAN^®^ 649 and the particle size and polydispersity index obtained. In addition, the concentration of the surfactant also showed an inverse relationship with the particle size obtained. The zeta potential was the least limiting factor of the dependent variables studied, resulting in values greater than /20/mV for all points studied. However, a directly proportional relationship was found between the interaction of the two independent variables and ZP. The optimized formulation had a particle size <200 nm and a polydispersity index <0.2, while the zeta potential presented was around −30 mV, maintaining these values for a month from the day of production and revealing the potential of this design. The reported data demonstrate the potential use of LN formulations and their optimization in the production of nutraceuticals, showing their ability to efficiently load the microalga extract [56,57], which is related to the growing interest in using LN to improve nutraceutical delivery and efficacy. Further studies are needed, though, to ensure the safety of the formulations and to study the kinetics of LN release in gastric simulation models or in vivo studies in order to confirm the potential to modify the release of loaded microalgae extract, as shown here in the rheological and differential scanning calorimetry analyses.

## Figures and Tables

**Table 1:** Upper, middle, and lower levels for each independent factor of the experimental design.

Independent Variable	Low Level (−1)	Medium Level (0)	Upper Level (+1)
% Tween^®^ 80	0.1	0.5	1
% SOFTISAN^®^ 649	1	2.5	3

**Table 2:** Physicochemical characteristics, namely, z-Ave, PI, and ZP, of LN over 30 days.

Formulation	Mean Particle Size (z-Ave, nm)			Polydispersity Index (PI)			Zeta Potential (mV)		
	Day 0	Day 14	Day 30	Day 0	Day 14	Day 30	Day 0	Day 14	Day 30
LN1	268.5 ± 0.59	273.3 ± 0.63	379.1 ± 1.30	0.172 ± 0.03	0.189 ± 0.04	0.282 ± 0.08	−24 ± 0.01	−22 ± 0.08	−20 ± 0.06
LN2	474.2 ± 1.15	489.5 ± 0.89	550.7 ± 0.91	0.299 ± 0.05	0.310 ± 0.06	0.358 ± 0.07	−44 ± 0.23	−44 ± 0.07	−38 ± 0.07
LN3	380.1 ± 0.86	392.8 ± 2.03	562.6 ± 0.68	0.253 ± 0.07	0.278 ± 0.03	0.319 ± 0.08	−37 ± 0.06	−35 ± 0.05	−34 ± 0.05
LN4	462.9 ± 0.27	478.1 ± 1.27	485.8 ± 0.73	0.374 ± 0.06	0.446 ± 0.01	0.523 ± 0.06	−34 ± 0.08	−33 ± 0.13	−34 ± 0.27
LN5	413.3 ± 0.77	426.6 ± 0.54	434.9 ± 1.24	0.05 ± 0.01	0.05 ± 0.02	0.08 ± 0.02	−33 ± 0.07	−30 ± 0.28	−30 ± 0.41
LN6	180.6 ± 1.10	190.2 ± 0.88	210.5 ± 1.15	0.126 ± 0.02	0.126 ± 0.01	0.146 ± 0.03	−38 ± 0.12	−39 ± 0.09	−38 ± 0.03

## Data Availability

The data used to support the findings of this study can be made available by the corresponding author upon request.
